# The impact of intelligent manufacturing strategy on enterprise labor productivity: evidence from a quasi-natural experiment in China

**DOI:** 10.3389/frai.2026.1775528

**Published:** 2026-05-26

**Authors:** Mingli Chen, Han Xu, Fa Tian, Li Ji

**Affiliations:** 1 School of Economics and Management, Jiangsu College of Engineering and Technology, Nantong, China; 2 School of Information Engineering, Jiangsu College of Engineering and Technology, Nantong, China; 3 School of Management, University of Shanghai for Science and Technology, Shanghai, China

**Keywords:** empirical analysis, innovation effect, intelligent manufacturing strategy, labor productivity, triple mediating pathways

## Abstract

**Introduction:**

Enhancing labor productivity is a critical driver for promoting the transformation and upgrading of the manufacturing sector.

**Methods:**

Utilizing data from intelligent manufacturing enterprises among Chinese listed companies, this study empirically examines the effects of China’s intelligent manufacturing strategy on enterprise labor productivity.

**Results:**

The results demonstrate that the strategy significantly enhances the labor productivity of intelligent manufacturing enterprises, and this conclusion remains robust after a series of tests, including parallel trend analysis, placebo tests, and propensity score matching. Further mechanism analysis reveals that the strategy improves labor productivity through three key channels: (1) fostering enterprise innovation, (2) optimizing human capital structure, and (3) increasing capital investment. Heterogeneity analysis indicates that the policy impact is more pronounced in the eastern and central regions, large-scale enterprises, and industries with slower technological iteration.

**Discussion:**

This study not only elucidates the mechanisms through which China’s intelligent manufacturing strategy enhances productivity but also provides valuable insights for other countries and regions implementing similar strategies

## 1 Introduction

Against the backdrop of profound restructuring in the global economic landscape and rapid iteration of digital technologies, labor productivity has evolved beyond merely a micro-level indicator of firm production efficiency. It has become a core determinant of corporate survival resilience and the quality of national economic growth. Existing studies have documented that improvements in labor productivity stem not from a single factor, but from the combined effects of multiple forces, including technological innovation, organizational restructuring, human resource management, and institutional environments (Gibson and Shrader, 2018; Bjuggren, 2018; Ye et al., 2023; Yang and Zhao, 2023; Xing et al., 2025).

The intelligent manufacturing strategy is a development strategy based on the in-depth integration of a new generation of information and communication technologies with advanced manufacturing technologies. It holds great significance for enterprises to promote industrial transformation and upgrading. By applying technologies such as artificial intelligence, big data, and cloud computing, intelligent manufacturing endows the manufacturing process with intelligent and automated capabilities (Qi and Xiao, 2020). Through customized production, it can meet consumers’ personalized needs (Mikalef et al., 2020), drive enterprises’ green technological innovation, and contribute to ecological environment improvement (Shen and Zhang, 2023; Bendig et al., 2023).

The application of new technologies not only cuts labor costs and improves equipment utilization but also significantly boosts labor productivity through carriers like industrial robots (Hecht, 2018; Graetz and Michaels, 2018; Tang et al., 2025). An in-depth investigation into how intelligent manufacturing policies affect labor productivity and the underlying mechanisms can provide important policy implications for China and other economies to seize emerging development opportunities in the digital era and promote high-quality economic growth.

In 2015, China released Made in China 2025, which identified intelligent manufacturing as the key priority. The paper treats the 2015 intelligent manufacturing strategy as a quasi-natural experiment, and systematically investigates the impact on labor productivity by using data from non-financial intelligent manufacturing listed companies on the Shanghai and Shenzhen A-shares. Compared with existing literature, the marginal contributions of this paper are mainly reflected in the following aspects:

Existing literature primarily focuses on how factors such as income tax exemptions and reductions (Yang et al., 2024), value-added tax (VAT) credit refunds (Sun et al., 2020; Wang and Li, 2024; Gu et al., 2025), social security investment (Liu et al., 2021), and the development of digital economy technologies (Li et al., 2023; Xing et al., 2025) affect firms’ labor productivity by shaping human capital endowments. However, relatively few studies have evaluated the effectiveness of external policies-such as intelligent manufacturing policies-and their underlying mechanisms of action. Building on this research gap, this study systematically investigates whether the 2015 intelligent manufacturing policy improves labor productivity and explores its potential transmission channels, thereby providing a valuable supplement to the existing literature.

This study systematically examines the impact of intelligent manufacturing policies on firms’ labor productivity, explores the underlying mechanisms. Our research findings reveals that intelligent manufacturing strategies effectively boost corporate innovation, human capital upgrading, innovation outputs and labor productivity. This study offers firm-level evidence from a major developing economy, enriches the literature on the dynamic determinants of firm-level labor productivity (Acemoglu and Restrepo, 2018; Autor et al., 2020), provides consistent empirical evidence for the economic effects of institutional reforms promoting intelligent manufacturing strategies, and delivers valuable policy implications and reform experiences for other emerging market and developing economies pursuing similar industrial upgrading.

The remainder of the paper proceeds as follows. Section 2 presents theoretical analysis and research hypotheses; section 3 describes research design and main data sources; section 4 reports empirical results and analysis; section 5 presents robustness tests; section 6 discuss mechanism test of action; section 7 reports further discussion. Finally, Section 8 concludes.

## 2 Theoretical analysis and research hypotheses

### 2.1 Theoretical analysis

Given the importance of labor productivity, existing studies focus on policy and institutional environment, enterprise management characteristics and technological upgrading. Overall, existing research mainly unfolds from two dimensions: external environmental factors and internal characteristic factors of enterprises.

#### 2.1.1 Regarding the impact of enterprises’ external environmental factors

At the policy and institutional level, tax incentive policies (Sun et al., 2020; Yang et al., 2024; Gu et al., 2025), consumption tax expectations (Zhang and Zhou, 2025), pre-tax deduction of employee education expenses (Ye et al., 2023), and increased public service expenditures (Jia and Si, 2025) all contribute to promoting enterprises’ labor productivity. Improving the legal environment (Li et al., 2021), enhancing enterprises’ compliance management (Shi et al., 2025), and increasing the flexibility of the labor market (Bjuggren, 2018) are beneficial to improving enterprises’ labor productivity. From the perspective of labor protection, trade unions, the minimum wage system, and the Labor Contract Law all promote enterprises’ labor productivity (Kim et al., 2020; Li and Zhao, 2020; Ku, 2022).

At the socio-economic environment level, the optimization of business environment (Niu et al., 2023), industrial agglomeration (Du et al., 2020), enterprises’ toward specialization transformation, refinement, characteristic development, innovation (Du et al., 2025), and supply chain technology regulations all contribute to promoting enterprises’ labor productivity. However, environmental pollution (He et al., 2019; Chen and Zhang, 2020) and financing constraints (Motta, 2020) are not conducive to labor productivity. The growth of local government public debt inhibits enterprises’ labor productivity (Wang and Zhao, 2022). Some studies have discussed the impact of transportation infrastructure such as airports and high-speed railways on labor productivity (Murakami and Kato, 2020).

#### 2.1.2 Regarding the impact of enterprises’ internal characteristic factors

At the enterprise management level, reverse mixed-ownership reform (Huang and Zhao, 2025), management efficiency (Zhang et al., 2024), employee co-investment (Cao and Chen, 2025), and management incentives (Gosnell et al., 2020; Kim et al., 2020) can exert a positive effect on enterprises’ labor productivity.

At the enterprise technological innovation level, enterprise technological progress (Kromann et al., 2020; Jin et al., 2025), digital transformation (Li and Jian, 2025), the utilization of data assets and data factors (Zhang et al., 2025; Zhu et al., 2025), the advancement of financial technology (Wu et al., 2025),the use of industrial robots (Hecht, 2018; Graetz and Michaels, 2018; Yu and Niu, 2025), and the application of artificial intelligence exerts a positive impact on labor productivity (Qi and Cheng, 2025). The improvement in the digitalization level boosts the labor productivity of upstream and downstream enterprises (Luo, 2025).

At the enterprise human resources level, health programs (Gubler et al., 2018), social security input (Zhang et al., 2023), labor quality (Liu and Wang, 2021), and intergenerational occupational mobility (Ji and Zhang, 2021) can all promote labor productivity.

Many scholars have explored the industrial policy effects of intelligent manufacturing policies (Wang and Dong, 2023; Li et al., 2025), and the impacts on enterprise innovation (Cao and Zeng, 2025), total factor productivity (TFP) (Shen et al., 2024), and new-quality productive forces (An, 2025). However, there is no relevant literature studying the impact on enterprise labor productivity. Exploring the inherent relationship between intelligent manufacturing policies and labor productivity is not only conducive to objectively evaluating the micro-level effects of the policy, but also can provide valuable policy insights for how to advance the implementation of the strategy.

The intelligent manufacturing strategy can enhance labor productivity through three channels. First, the financial support provided by a series of supporting policies helps relieve firms’ internal and external resource constraints, incentivizes them to increase R&D investment, and thus provides innovation support for improving labor productivity; Second, intelligent manufacturing policies are conducive to upgrading firms’ human capital structure. The expansion of high-level human capital helps improve resource allocation efficiency and innovation capacity, offering a human capital channel for enhancing labor productivity; Third, the alleviation of financing constraints encourages firms to acquire high-end intelligent equipment and intelligent software, increases capital input and improves the hardware resources required for innovation, thereby providing material capital support for the improvement of labor productivity.

Based on the above analysis, we propose Hypothesis 1: Intelligent manufacturing strategy is conducive to improving firms’ labor productivity.

### 2.2 Analysis of the mechanism of action

#### 2.2.1 Based on the enterprise innovation mechanism: alleviating enterprise financing constraints and enhancing enterprise innovation level

The intelligent manufacturing strategy can alleviate the financing constraints by enhancing enterprises’ own quality and optimizing the external financing environment through paths such as the integration of industry and finance, digital empowerment, and policy guidance. The specific impact logic is as follows:

At the level of industrial and financial integration, the strategy facilitates the deep integration between intelligent manufacturing firms and the financial sector. On the one hand, when firms implement the strategy, their production and operational data can be disclosed through platforms such as the industrial internet. Financial institutions can use these data to accurately assess firms’ operating conditions and creditworthiness, supporting the development of businesses including supply chain finance (Zhang and Yang, 2025). For instance, financial institutions can provide financing based on firms’ transaction data in the supply chain, mitigating information asymmetry (Xiao et al., 2024), lowering the risk perceptions of financial institutions, and improving firms’ access to credit. On the other hand, intelligent manufacturing policies promote the large-scale adoption of industrial robots, which effectively reduces production costs (Yao et al., 2024). This enables firms to aggregate and optimize resources efficiently, thereby alleviating their capital pressure (Wang and Dong, 2023).

At the level of digital empowerment, improved digitalization enhances firms’ financial conditions and operational stability, making it easier for them to gain recognition from financial institutions. Meanwhile, digitalization can optimize firms’ financing processes: by leveraging financial technology, enterprises can submit financing applications and supporting documents more conveniently, and financial institutions can conduct reviews and disburse funds more efficiently. In addition, digitalization improves firms’ information transparency and mitigates information asymmetry between investors and firms, thereby attracting more investors. Increased cooperation and collaborative innovation also help firms obtain more trade credit support.

At the level of policy guidance, both the central and local governments have introduced a series of supportive policies. Tax incentive policies can reduce firms’ financial burdens, enabling them to allocate more funds to R&D and production-thereby enhancing firms’ profitability and debt-servicing capacity. Special funds established by the government, such as the National Special Fund for Intelligent Manufacturing, focus on supporting the digital transformation of small and medium-sized enterprises (SMEs), providing direct financial support and thus easing firms’ capital pressure (Yin and Li, 2022). Additionally, the government can establish risk compensation mechanisms and cooperate with financial institutions to share lending risks. This reduces the credit risks of financial institutions and incentivizes them to provide more financial support to firms (Shen et al., 2024).

R&D investment, however, is characterized by high risk, high uncertainty, long cycles, and strong asset specificity (An et al., 2020). Essentially, enterprise R&D investment is a capital-intensive activity that requires continuous capital flow support. The essence of financing constraints lies in insufficient external capital supply or limited internal capital accumulation, which prevents the filling of capital gaps required for R&D and ultimately inhibits R&D investment either directly or indirectly. The financing constraint alleviation effect of intelligent manufacturing policies can provide financial support for enterprises’ R&D and innovation. This encourages enterprises to increase R&D investment, enhance their innovation level, and ultimately improve their labor productivity.

R&D investment, however, is characterized by high risk, high uncertainty, long cycles, and strong asset specificity (An et al., 2020). Essentially, firm R&D investment is a capital-intensive activity that requires sustained capital flow support. The financing constraint mitigation effect of intelligent manufacturing policies can provide financial support for firms’ R&D and innovation activities. This, in turn, incentivizes firms to increase R&D investment, enhance their innovation capacity, and ultimately improve their labor productivity.

Based on the above analysis, we propose Hypothesis 2: Intelligent manufacturing strategy improves firms’ labor productivity by alleviating financing constraints, thereby enhancing their innovation capacity.

#### 2.2.2 At the level of human capital upgrading: enhancing enterprises’ human capital level

The strategy can optimize human capital structure of enterprises and improve the efficiency of human resource utilization. In the digital era, human capital accumulation has become a crucial source for enterprises to gain core competitiveness. The intelligent manufacturing strategy creates an advanced technological and innovative environment, which can attract more high-education, high-quality innovative talents (Goldfarb et al., 2023). Meanwhile, enterprises will strengthen the training of internal employees, improve employees’ knowledge and skills, enhance their ability to master advanced technologies-providing talent guarantees and intellectual support for enterprise innovation (Yin and Li, 2022).

The strategy involves significant investment in fixed assets such as intelligent machinery and electronic equipment. These devices have a notable substitution effect on unskilled labor, displacing some unskilled jobs and making “machine replacement of labor” a major trend in the manufacturing industry (Acemoglu and Restrepo, 2020). This drives enterprises to hire more high-skilled talents, thereby enhancing the overall level of human capital (Yao et al., 2024). Furthermore, industries with rapid technological iteration in promoting the optimization of enterprises’ human capital structure is more prominent-enterprises will continuously invest resources to improve labor quality and attract the concentration of more high-skilled talents (Goldfarb et al., 2023).

Based on the above analysis, we propose Hypothesis 3: Intelligent manufacturing strategy enhances firms’ labor productivity by facilitating human capital upgrading.

#### 2.2.3 Based on the capital input mechanism: enhancing the level of capital input

High-end intelligent equipment is characterized by high unit prices and long investment payback periods, so enterprises’ willingness to make initial investments is easily constrained by costs. Intelligent manufacturing policies stimulating their motivation for capital investment. The government provides direct financial support to purchase high-end intelligent equipment and build intelligent production lines by establishing special funds (such as the National Intelligent Manufacturing Special Fund and local digital transformation subsidies) (Quan and Li, 2022). On the one hand, the “accelerated depreciation of fixed assets” policy is implemented (Liu et al., 2025), allowing enterprises to accrue higher depreciation amounts for high-end intelligent equipment in the short term. On the other hand, tariff reductions are applied to the import of intelligent manufacturing-related equipment, or tax credits are offered for enterprises’ technological transformation investments. When intelligent manufacturing enterprises increase their investment in high-end equipment, they can improve the skill levels of their high-skilled talents, enhance the efficiency of internal resource collaboration and resource allocation (Wen et al., 2025), and at the same time replace low-end repetitive work (Acemoglu and Restrepo, 2020).

Based on the above analysis, we propose Hypothesis 4: Intelligent manufacturing strategy improves firms’ labor productivity by facilitating their equipment upgrading.

**Figure 1:**
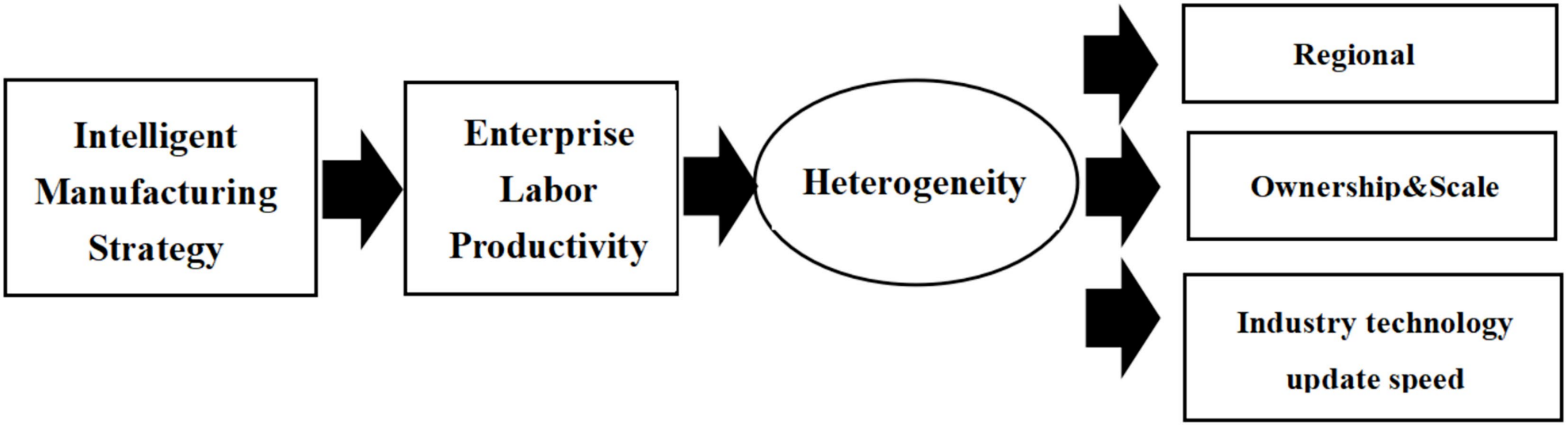
Mechanism framework: the conceptual framework linking the intelligent manufacturing strategy to enterprise labor productivity.

Building on the theoretical analysis, the direction and interaction of these mechanisms require empirical validation. Figure 1 presents the conceptual framework that links intelligent manufacturing strategy to enterprise labor productivity.

## 3 Research design

### 3.1 Specification of the econometric model

To examine the impact of the 2015 intelligent manufacturing strategy on enterprise labor productivity, this paper employs a sample of Chinese A-share listed firms on the Shanghai and Shenzhen Stock Exchanges. The sample is classified into two groups: intelligent manufacturing enterprises and non-intelligent manufacturing enterprises. Following the approach of prior studies (Huang et al., 2023), we construct a difference-in-differences (DID) Equation 1 to conduct the empirical regression analysis:

$${\mathrm{MPL}}_{\mathrm{it}}=\alpha_{0}+\theta {\text{Treat}}_{i}\times {\text{Post}}_{t}+\beta X_{\mathrm{it}}+\mu_{i}+\gamma_{t}+\varepsilon_{\mathrm{it}}$$ (1)

Among them, the subscripts i and t represent the enterprise and year, respectively. The explained variable MPL denotes the labor productivity of enterprise i in year t. The core variable Treat is defined as follows: enterprises whose industry falls into the 10 key supported fields specified in the Made in China 2025 industrial policy are identified as samples of intelligent manufacturing enterprises, in which case Treat equals 1; otherwise, it equals 0. Post equals 1 if the year is greater than or equal to 2015, and 0 otherwise.

The core competitiveness of intelligent manufacturing enterprises stems from technological innovation (e.g., industrial software, intelligent equipment, and algorithm research and development). Their R&D investment as a percentage of operating revenue is typically 2–3 times that of ordinary manufacturing enterprises (according to the Made in China 2025 white paper). The core of the additional deduction policy for R&D expenses is to grant tax reductions in proportion to R&D investment: the higher the R&D input, the greater the tax reduction. The policy significantly eases corporate cash flow pressures and financing constraints, thereby further encouraging enterprises to expand R&D and improve performance.

Therefore, compared with general manufacturing enterprises, the impact of the R&D policy is more pronounced for intelligent manufacturing enterprises (Mukherjee et al., 2017; Li et al., 2023).

The policy variable of interest in this paper is the interaction term Treat×Post, where Treat is the treatment variable and Post is the policy shock variable. θ is the regression coefficient that this paper focuses on primarily. If the estimated coefficient θ is positive, it indicates that the intelligent manufacturing policy is conducive to improving the labor productivity. This paper also controls for a series of variables related to enterprise-level characteristics, as well as enterprise fixed effects ( $\mu_{i}$ ) and time fixed effects ( $\gamma_{t}$ ).

### 3.2 Variable definition

Enterprise labor productivity (MPL). For the measurement of enterprise labor productivity (MPL), drawing on existing studies (Bender et al., 2018), which mainly use the unit output of company employees for measurement, we therefore measure it using the natural logarithm of the ratio of listed companies’ operating income to the number of company employees.

To mitigate potential confounding effects on the empirical results, this study selects a set of firm-level control variables. Following prior literature, the control variables are defined as follows: firm age (age), measured as the natural logarithm of the number of years since the firm’s founding; return on assets (roa), calculated as net income divided by total assets; firm leverage (lev), measured as the ratio of year-end total liabilities to total assets; operating cash flow (cash), computed as net cash flow from operating activities divided by total assets; firm growth (growth), measured by the annual growth rate of operating revenue; capital intensity (kdensity), defined as the natural logarithm of fixed assets per employee; firm value (tbq, Tobin’s Q); management ownership (mshare), measured as the proportion of shares held by management; and the ownership concentration (bigholder), represented by the shareholding ratio of the largest shareholder.

### 3.3 Sample selection and descriptive statistics of variables

This study employs a sample of Chinese manufacturing listed firms on the Shanghai and Shenzhen A-share markets from 2010 to 2020. Firm-level characteristics and financial data are mainly obtained from the WIND and CSMAR (China Stock Market and Accounting Research) databases. The sample period is selected for two reasons. First, the official implementation of the intelligent manufacturing policy in 2015 allows a 5-year window before and after the policy shock, which not only ensures sufficient sample size but also mitigates potential policy confounding effects associated with an overly long time span. Second, considering the potential disruptions caused by the COVID-19 pandemic, the sample period ends in 2020.

Following standard practices in the literature, we clean the raw data to ensure data quality and reliability: (1) We restrict the sample to manufacturing listed companies and exclude firms in the financial and other non-manufacturing industries; (2) We eliminate firms with a short listing history and those with missing values in key financial variables; (3) To alleviate the influence of outliers, all continuous variables are winsorized at the 1st and 99th percentiles. Descriptive statistics for the main variables are presented in Table 1.

**Table 1:** Descriptive statistics of main variables.

VarName	Obs.	Mean	SD	Min	Max
MPL	16,077	21.425	1.328	18.811	25.236
High_edu	16,336	0.219	0.166	0.000	0.783
Skills	15,709	0.174	0.119	0.000	0.618
Rdperson	12,781	0.129	0.108	0.000	0.545
Devices	10,444	19.311	1.625	15.177	23.357
Treat	16,846	0.702	0.457	0.000	1.000
Post	16,846	0.660	0.474	0.000	1.000
Age	16,846	2.840	0.329	1.792	3.466
Roa	16,077	0.046	0.052	−0.148	0.196
Lev	16,846	0.396	0.192	0.052	0.860
Cash	16,077	0.051	0.065	−0.129	0.234
Growth	16,077	11.422	25.731	−60.275	113.156
Kdensity	15,578	12.610	0.842	10.355	14.726
Tbq	16,516	2.126	1.299	0.891	8.357
Mshare	16,506	12.999	19.817	0.000	68.596
Bigholder	16,510	0.341	0.142	0.029	0.900

## 4 Empirical results and analysis

### 4.1 Intelligent manufacturing policy and labor productivity: baseline regression

To examine the impact of the strategy on enterprises’ labor productivity, this paper uses the aforementioned (Equation 1) to test the sample data, and the regression results are presented in Table 2. In the regression results of Table 2, Column (1) only includes the interaction term (Treat×Post). It can be observed that the estimated coefficient of the interaction term (Treat×Post) is 0.194, and it passes the significance test at the 1% level. This indicates that the 2015 intelligent manufacturing strategy can significantly improve enterprises’ labor productivity, verifying the conclusion of this paper.

**Table 2:** Baseline regression.

Variables	(1)	(2)	(3)	(4)
	MPL	MPL	MPL	MPL
Treat_post	0.194***	0.141***	0.138***	0.153***
	(0.031)	(0.027)	(0.027)	(0.026)
Age		0.844***	0.831***	0.908***
		(0.133)	(0.136)	(0.138)
Lev		1.356***	1.337***	1.208***
		(0.083)	(0.085)	(0.076)
Cash		0.835***	0.806***	0.708***
		(0.081)	(0.082)	(0.075)
Roa		2.066***	2.075***	2.280***
		(0.133)	(0.134)	(0.130)
Kdensity			0.006	0.015
			(0.019)	(0.016)
Mshare			−0.002***	−0.002***
			(0.000)	(0.000)
Bigholder			0.043	−0.112
			(0.153)	(0.136)
Growth				0.006***
				(0.000)
Tbq				−0.034***
				(0.006)
YEAR	YES	YES	YES	YES
IND	YES	YES	YES	YES
Constant	21.663***	18.813***	18.866***	18.488***
	(0.018)	(0.336)	(0.420)	(1.007)
Observations	16,077	16,077	15,569	15,244
R-squared	0.369	0.465	0.465	0.547
Number of stkcd	2,423	2,423	2,350	2,348

The table reports baseline regression of intelligent manufacturing policy and labor productivity. ***, **, * denote the significance of there spective coefficients at 1, 5, and 10% levels.

To control for biases in the regression results caused by omitted variables, we gradually add firm-level characteristic control variables in Columns (2), (3), and (4). It can be found that as we include the control variables, the coefficient of the interaction term (Treat×Post) remains positive and all pass the significance test at the 1% level. The regression results still support the aforementioned conclusion.

## 5 Robustness tests

To further verify the robustness of the baseline regression results, we perform a battery of robustness checks across multiple dimensions, including the parallel trend test, placebo test, propensity score matching (PSM) analysis, and controlling for R&D intensity.

### 5.1 Parallel trend test

An important assumption for the validity of the difference-in-differences (DID) model in application is that the treatment group and the control group satisfy the parallel trend. Specifically, in this study, it requires that without the impact of the 2015 intelligent manufacturing policy, the changing trends of labor productivity between the treatment group enterprises and the control group enterprises should be parallel. If the parallel trend is not satisfied, the estimation results of the DID model may be biased. To address this issue, we draw on the practices of existing scholars (Chen et al., 2018) and adopt an event study approach based on Equation 1 to construct a more flexible dynamic regression for testing the parallel trend. We also identify the timing of the policy effect, taking the year before the policy was introduced (2014) as the base year. On the basis of Equation 1, we construct the following econometric Equation 2:

$${\mathrm{MPL}}_{\mathrm{it}}=\alpha_{0}+\sum_{2010}^{2020}\theta_{t}{\text{Treat}}_{i}\times {\text{Year}}_{t}+\beta X_{\mathrm{it}}+\mu_{i}+\gamma_{t}+\varepsilon_{\mathrm{it}}$$ (2)

In the above equation, ${\text{Year}}_{t}$ represents a year-by-year dummy variable; $\theta_{t}$ reflects the relative impact of the intelligent manufacturing policy on the labor productivity in the treatment group in year t. If the parallel trend assumption is satisfied before the policy implementation, then the changes in enterprises’ labor productivity after 2015 are the policy effects resulting from the implementation of the intelligent manufacturing policy. Other variables in Equation 2 are consistent with those defined earlier.

**Figure 2:**
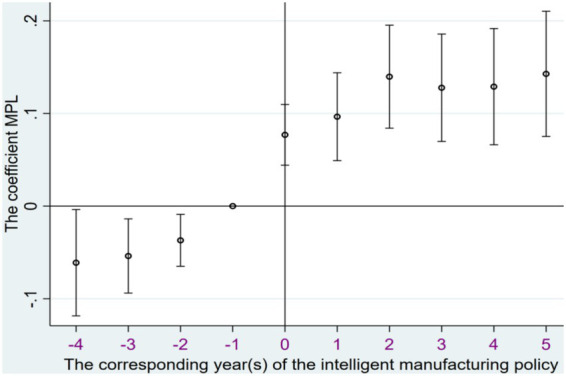
Parallel trend test.

We perform a regression analysis based on Equation 2. Figure 2 reports the annual regression coefficients of the interaction term and illustrates the dynamic parallel trend plot. As shown in Figure 2, no significant difference in labor productivity changes exists between the treatment and control groups prior to policy implementation, indicating that the parallel trend assumption is satisfied. Following the implementation of the intelligent manufacturing policy in 2015, a significant divergence between the two groups emerges. The policy effect peaks 3 years after implementation and remains significant for several consecutive years.

### 5.2 Placebo test

To address potential biases driven by external random factors, this study conducts a placebo test following the approach of La Ferrara et al. (2012) to verify that our baseline results are not driven by spurious random events. Specifically, we artificially randomize the assignment of the treatment and control groups to construct a pseudo-policy shock, and then re-estimate the baseline model using this simulated sample.

**Figure 3:**
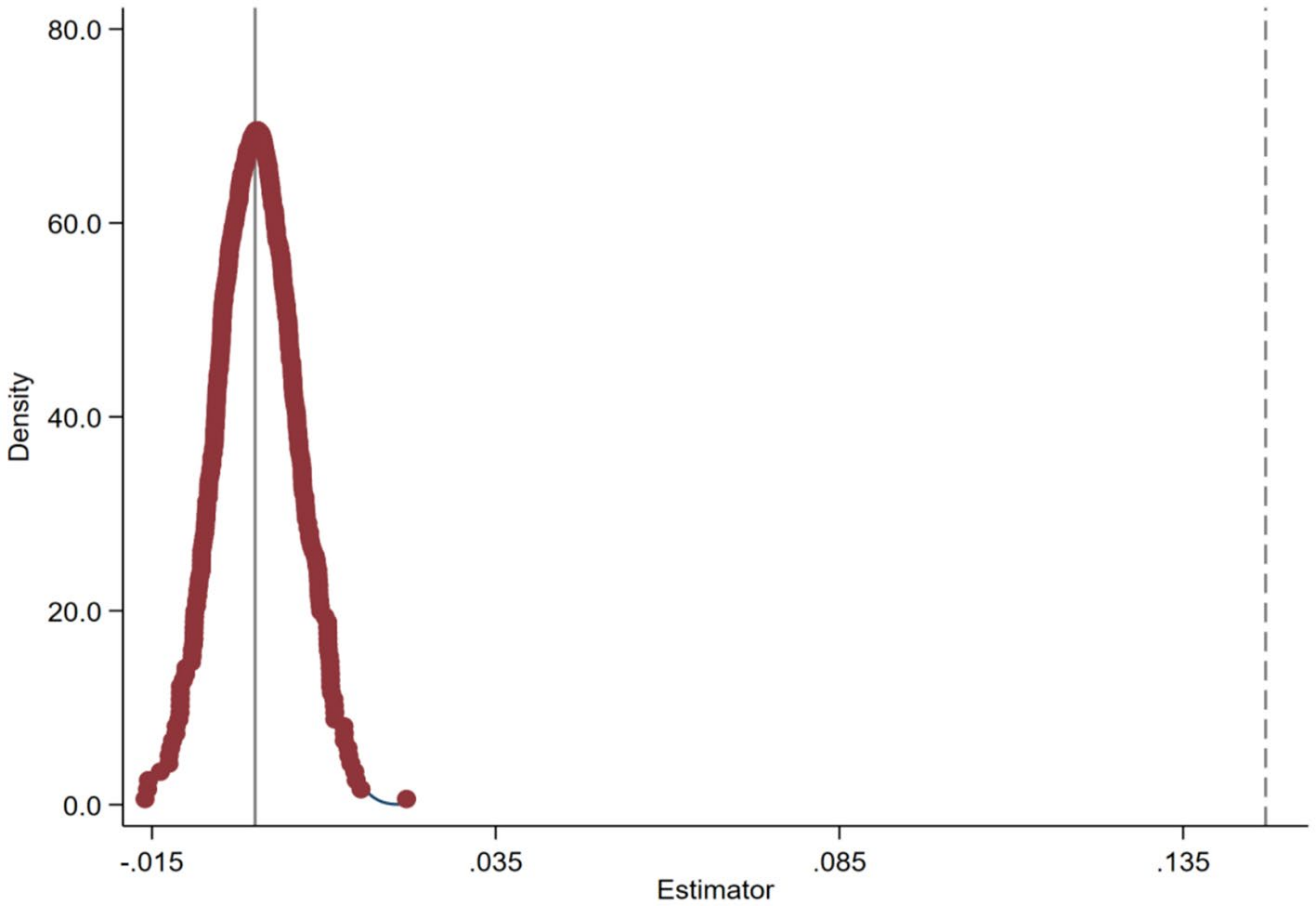
Placebo test.

Figure 3 presents the probability density distribution of the estimated coefficients from 500 random permutations of the pseudo-policy shock. As shown, most of the estimated coefficients from the placebo regressions are centered around zero and follow a normal distribution. In contrast, our baseline coefficient (0.147) lies distinctly on the right side of zero, implying that the randomly simulated shocks exert no significant effect on enterprise labor productivity. The placebo test confirms that the observed policy effect is not attributable to random factors, but instead reflects a genuine causal impact. Collectively, these findings alleviate concerns over time-invariant confounding factors and further validate that the positive effect of intelligent manufacturing policies on firm labor productivity is robust and credible.

### 5.3 Propensity score matching

Before the implementation of the intelligent manufacturing policy, if there are significant characteristic differences between enterprises in the treatment group and the control group, it will interfere with the regression results of the difference-in-differences (DID) model. To verify the interference of such significant differences on the regression results, we adopt the Propensity Score Matching (PSM) method to identify the control group with similar characteristics. We use the control variable X as the covariate and adopt the 1:5 nearest neighbor matching method to select the control group.

Column (2) in Table 3 reports the matching results based on the Logit model, while Columns (3)–(6) present the balance test of covariates, which verifies the effectiveness of the PSM method. The results show that the covariate differences between the treatment group and the control group decrease significantly after matching. We conduct the DID test based on the data after PSM matching. From the regression results, we can find that the regression coefficient of Treat×Post remains positive and passes the significance test at the 1% level, which verifies the baseline regression results.

**Table 3:** Regression results of propensity score matching (PSM).

Panel A: PSM process						Panel B: DID estimation	
Variables	Logit model	Covariate balance test				Variables	MPL
		Matched status	Treatment group	Control group	Bias		
Age	−0.610***	NO	2.840	2.862	−6.8	Treat×Post	0.137*
	(0.141)	YES	2.840	2.843	−1		(0.0279)
Lev	−0.203**	NO	0.385	0.404	−10	Control Var	YES
	(0.112)	YES	0.385	0.384	0.3	Year	YES
Cash	−3.438***	NO	0.048	0.059	−17	Ind	YES
	(0.318)	YES	0.048	0.047	0.8	Sample Size	13,017
Roa	0.907	NO	0.047	0.046	2.5	adj_R2	0.542
	(0.420)	YES	0.047	0.048	−1.1		
Kdensity	−0.136***	NO	12.571	12.703	−15.4		
	(0.023)	YES	12.571	12.551	2.4		
Mshare	0.001	NO	13.869	12.225	8.2		
	(0.001)	YES	13.869	13.955	−0.4		
Bigholder	−1.144***	NO	0.333	0.359	−19		
	(0.131)	YES	0.333	0.335	−1.5		
Growth	0.003***	NO	11.782	9.809	7.9		
	(0.001)	YES	11.782	11.171	2.5		
Tbq	0.112***	NO	2.129	1.967	13.3		
	(0.018)	YES	2.129	2.100	2.4		
Constant term	2.443***						
	(0.540)						
Sample size	15,039						
Pseudo R-squared	0.023						

The table reports regression results of propensity core matching (PSM). ***, **, * denote the significance of the respective coefficients at 1, 5, and 10% levels.

### 5.4 Increasing R&D expense intensity

The intelligent manufacturing policy explicitly emphasizes an innovation-driven development strategy, and R&D intensity serves as a key indicator of such innovation orientation. The policy aims to support the development of intelligent manufacturing firms, incentivize them to raise R&D investment, and enhance their innovation capabilities. Accordingly, we expect that firms with higher R&D intensity are likely to be more responsive to the policy. In this section, we construct an interaction term involving enterprise R&D intensity (Treat×Post×RD_invest) to examine the policy’s impact on enterprises with high R&D intensity.

From the regression results in Table 4, it can be observed that the coefficients of the R&D intensity interaction term (Treat×Post×RD_invest) are all relatively positive and basically significant at the 1 and 5% levels. This further verifies the robustness of the conclusions.

**Table 4:** Test results of increasing R&D expense intensity.

Variables	(1)	(2)	(3)	(4)
	MPL	MPL	MPL	MPL
Treat×post×RD_invest	0.030***	0.026***	0.027***	0.018*
	(0.011)	(0.010)	(0.010)	(0.010)
Treat×RD_invest	−0.068***	−0.054***	−0.053***	−0.037***
	(0.007)	(0.006)	(0.006)	(0.006)
Post×RD_invest	−0.010	−0.017*	−0.016*	−0.006
	(0.009)	(0.009)	(0.009)	(0.009)
Treat_post	0.105**	0.083*	0.083*	0.107**
	(0.046)	(0.043)	(0.042)	(0.042)
Age		0.739***	0.716***	0.779***
		(0.143)	(0.140)	(0.146)
Lev		1.101***	1.247***	1.121***
		(0.085)	(0.085)	(0.078)
Cash		0.713***	0.680***	0.639***
		(0.084)	(0.080)	(0.077)
Roa			2.084***	2.282***
			(0.141)	(0.143)
Kdensity			−0.005	0.007
			(0.019)	(0.017)
Mshare			−0.002***	−0.002***
			(0.000)	(0.000)
Bigholder				−0.211
				(0.155)
Growth				0.005***
				(0.000)
Tbq				−0.032***
				(0.006)
Year	Yes	Yes	Yes	Yes
Ind	Yes	Yes	Yes	Yes
Constant	20.968***	18.740***	18.701***	17.261***
	(0.035)	(0.361)	(0.417)	(1.107)
Observations	14,493	14,493	14,358	14,073
R-squared	0.425	0.480	0.508	0.575
Number of stkcd	2,331	2,331	2,301	2,299

The table reports test results of increasing R&D expense intensity. ***, **, * denote the significance of the respective coefficients at 1, 5, and 10% levels.

### 5.5 Regional digital development level

The level of regional digital development serves as a key indicator reflecting local informatization and intelligentization. Theoretically, regions with a higher level of digital development tend to enjoy a higher degree of intellectualization, thereby strengthening the effects of the intelligent manufacturing strategy. To verify the robustness of the baseline regression results, we draw on existing research (Huang et al., 2023) and use the regional digital development level^1^ (Internet) for the test. Intelligent manufacturing enhances enterprise labor productivity by improving firms’ intellectualization and informatization levels. Accordingly, we expect that firms located in regions with higher digital development are more responsive to the policy.

In this section, we construct a triple interaction term involving the regional digital development level (Treat × Post × Internet) to examine the policy’s impact. From the regression results in Table 5, it can be observed that the coefficients of the interaction term for regional digital development level (Treat × Post × Internet) are all relatively significant, which further verifies the robustness of the conclusions in this paper.

**Table 5:** Regression results of the test on regional digital development level.

Variables	(1)	(2)	(3)	(4)
	MPL	MPL	MPL	MPL
Treat×post×internet	0.133***	0.072**	0.072**	0.077**
	(0.038)	(0.034)	(0.033)	(0.032)
Treat_post	0.128***	0.104***	0.103***	0.112***
	(0.035)	(0.033)	(0.032)	(0.031)
Age		0.834***	0.812***	0.853***
		(0.150)	(0.146)	(0.150)
Lev		1.195***	1.344***	1.193***
		(0.096)	(0.095)	(0.085)
Cash		0.723***	0.685***	0.644***
		(0.095)	(0.091)	(0.082)
Roa			2.269***	2.568***
			(0.161)	(0.165)
Kdensity			−0.014	0.004
			(0.020)	(0.018)
Mshare			−0.001***	−0.002***
			(0.001)	(0.000)
Bigholder				−0.089
				(0.152)
Growth				0.005***
				(0.000)
Tbq				−0.053***
				(0.007)
Year	Yes	Yes	Yes	Yes
Ind	Yes	Yes	Yes	Yes
Constant	20.981***	18.418***	18.482***	16.351***
	(0.017)	(0.380)	(0.441)	(1.065)
Observations	12,561	12,561	12,414	12,113
R-squared	0.362	0.435	0.468	0.556
Number of stkcd	2,047	2,047	2,020	2,018

The table reports test results of regression results of the test on regional digital development level. ***, **, * denote the significance of the respective coefficients at 1, 5, and 10% levels.

## 6 Mechanism test of action

From the theoretical analysis in the previous sections, it can be concluded that the implementation of intelligent manufacturing policies guides enterprises in intelligent transformation, which optimizes the allocation of existing resources and improves the labor productivity. Therefore, the paper will further examine the aforementioned mechanisms below.

### 6.1 Innovation-driven dimension: alleviating enterprises’ financing constraints and enhancing enterprises’ innovation level as a channel

Our earlier theoretical analysis suggests that intelligent manufacturing policies can alleviate firms’ financing constraints through channels such as industry–finance integration, digital empowerment, and policy guidance. These policies not only improve the efficiency of internal capital allocation (Wang and Dong, 2023) but also facilitate access to external financing and government subsidies. By easing financial pressure, intelligent manufacturing policies enhance production and operational efficiency, while providing additional financial resources to strengthen innovation capacity. Ultimately, the improvements in resource allocation efficiency and technological innovation capability jointly promote the growth of enterprise labor productivity.

Following the approach of existing scholars (Ju et al., 2013), this paper uses the corporate financing constraint indices SA (finan_bind) and FC (FC) to measure the level of enterprises’ financing constraints. Based on Equation 1, the following Equations 3, 4 is constructed:

$$\text{Finan}\_{\text{bind}}_{\mathrm{it}}=\alpha_{0}+\theta {\text{Treat}}_{i}\times {\text{Post}}_{t}+\beta X_{\mathrm{it}}+\mu_{i}+\gamma_{t}+\varepsilon_{\mathrm{it}}$$ (3)

$${\mathrm{FC}}_{\mathrm{it}}=\alpha_{0}+\theta {\text{Treat}}_{i}\times {\text{Post}}_{t}+\beta X_{\mathrm{it}}+\mu_{i}+\gamma_{t}+\varepsilon_{\mathrm{it}}$$ (4)

The regression results are shown in Columns (1)–(4) of Table 6. It can be observed that intelligent manufacturing policies have a significant effect on alleviating enterprises’ financing constraints. This indicates that intelligent manufacturing policies alleviate enterprises’ financing constraints and improve their labor productivity. Meanwhile, in the regressions of Columns (5) and (6), we include government subsidies for intelligent manufacturing enterprises (subsidy). It can be found that government subsidies alleviate enterprises’ financing constraint levels to a certain extent.

**Table 6:** Mechanism test at the innovation-driven dimension: alleviating financing constraints.

Variables	(1)	(2)	(3)	(4)	(5)	(6)
	Finan_bind	Finan_bind	FC	FC	Finan_bind	FC
Treat_post	−0.010***	−0.007*	−0.049***	−0.020***	−0.003	−0.018**
	(0.004)	(0.003)	(0.009)	(0.007)	(0.004)	(0.008)
Treat_post_sub					−0.003***	−0.002
					(0.001)	(0.002)
Subsidy					0.001**	−0.003**
					(0.001)	(0.001)
Age		−0.075***		−0.211***	−0.073***	−0.210***
		(0.021)		(0.039)	(0.021)	(0.039)
Lev		−0.061***		−0.653***	−0.059***	−0.653***
		(0.008)		(0.021)	(0.008)	(0.021)
Cash		−0.016*		−0.012	−0.015*	−0.012
		(0.008)		(0.025)	(0.008)	(0.025)
Roa		−0.062***		−0.343***	−0.062***	−0.345***
		(0.014)		(0.037)	(0.015)	(0.037)
Kdensity		0.003		−0.027***	0.004*	−0.025***
		(0.002)		(0.005)	(0.002)	(0.005)
Mshare		0.000***		0.001***	0.000***	0.001***
		(0.000)		(0.000)	(0.000)	(0.000)
Bigholder		0.042**		0.064*	0.045**	0.080**
		(0.018)		(0.039)	(0.018)	(0.036)
Growth		−0.000***		−0.000***	−0.000***	−0.000***
		(0.000)		(0.000)	(0.000)	(0.000)
Tbq		0.006***		−0.014***	0.006***	−0.014***
		(0.001)		(0.002)	(0.001)	(0.002)
Year	Yes	Yes	Yes	Yes	Yes	Yes
Ind	Yes	Yes	Yes	Yes	Yes	Yes
Constant	−3.567***	−3.424***	0.648***	1.726***	−3.437***	1.695***
	(0.002)	(0.059)	(0.006)	(0.115)	(0.059)	(0.112)
Observations	16,064	15,244	16,496	15,244	15,088	15,088
R-squared	0.886	0.901	0.146	0.384	0.901	0.385
Number of stkcd	2,422	2,348	2,424	2,348	2,346	2,346

The table reports test results of mechanism test at the Innovation-Driven dimension: Alleviating Financing Constraints. ***, **, * denote the significance of the respective coefficients at 1, 5, and 10% levels.

The impact of intelligent manufacturing strategy on enterprises’ R&D investment and innovation level is shown in Table 7. From the regressions, it can be observed that the regression coefficients on enterprises’ R&D investment (rd_invest) and innovation level (total_patent) are both positive, and basically all pass the significance test at the 1% level.

**Table 7:** Mechanism test at the innovation-driven dimension: enhancing innovation level.

Variables	(1)	(2)	(3)	(4)	(5)	(6)
	Rd_invest	Rd_invest	Rd_invest	Total_patent	Total_patent	Total_patent
Treat_post	0.402***	0.455***	0.410***	0.128***	0.102***	0.099**
	(0.101)	(0.098)	(0.104)	(0.042)	(0.039)	(0.039)
Age		−1.622***	−1.799***		0.491***	0.596***
		(0.580)	(0.573)		(0.159)	(0.179)
Lev		−1.345***	−0.988***		0.372***	0.375***
		(0.289)	(0.284)		(0.085)	(0.087)
Cash		−2.497***	−1.929***		0.0680	0.080
		(0.389)	(0.375)		(0.079)	(0.090)
Roa		−1.507**	−1.783***		0.261*	0.347**
		(0.595)	(0.610)		(0.138)	(0.142)
Kdensity		0.144**	0.087		0.016	0.015
		(0.072)	(0.069)		(0.021)	(0.021)
Mshare		0.000	0.000		−0.000	−0.000
		(0.002)	(0.002)		(0.001)	(0.001)
Bigholder			−0.160			−0.223
			(0.565)			(0.197)
Growth			−0.015***			0.000
			(0.001)			(0.000)
Tbq			−0.049*			−0.029***
			(0.029)			(0.007)
Year	Yes	Yes	Yes	Yes	Yes	Yes
Ind	Yes	Yes	Yes	Yes	Yes	Yes
Constant	3.901***	6.815***	8.037**	3.347***	3.337***	4.160***
	(0.150)	(1.703)	(3.425)	(0.031)	(0.253)	(1.167)
Observations	14,677	14,358	14,073	16,561	15,355	15,039
R-squared	0.031	0.046	0.097	0.704	0.697	0.703
Number of stkcd	2,331	2,301	2,299	2,406	2,331	2,329

The table reports test results of mechanism test at the Innovation-Driven dimension: Enhancing Innovation Level. ***, **, * denote the significance of the respective coefficients at 1, 5, and 10% levels.

Based on the above analysis, this paper argues that the strategy alleviates enterprises’ financing constraints, enabling firms to allocate sufficient capital to innovation activities and capital upgrading, which in turn promotes the improvement of enterprise labor productivity (MPL).

### 6.2 Human capital upgrading dimension: enhancing enterprises’ human capital level

In the process of implementing intelligent manufacturing strategies, firms have both the incentive and the necessity to expand their demand for highly educated and highly skilled labor, thereby optimizing their human capital structure (Yin and Li, 2022). High-quality human capital not only promotes production and operational efficiency, enhances innovation capacity, and drives high-quality development, but also ultimately contributes to the improvement of enterprise labor productivity.

To verify the mechanism that intelligent manufacturing strategies affect enterprises’ innovation level by enhancing their human capital, this paper uses the proportion of employees with a bachelor’s degree or above (High_edu) and the proportion of technical personnel in listed companies (High_skills) to measure the upgrading of enterprises’ human capital structure. Based on Equation 1, the following Equations 5, 6 is constructed:

$$\text{High}\_{\mathrm{edu}}_{\mathrm{it}}=\alpha_{0}+\theta {\text{Treat}}_{i}\times {\text{Post}}_{t}+\beta X_{\mathrm{it}}+\mu_{i}+\gamma_{t}+\varepsilon_{\mathrm{it}}$$ (5)

$$\text{High}\_{\text{skills}}_{\mathrm{it}}=\alpha_{0}+\theta {\text{Treat}}_{i}\times {\text{Post}}_{t}+\beta X_{\mathrm{it}}+\mu_{i}+\gamma_{t}+\varepsilon_{\mathrm{it}}$$ (6)

As shown in the regression results in Table 8, intelligent manufacturing strategies significantly raise the share of employees with a bachelor’s degree or above, strengthen the employment of high-skilled talents, optimize firms’ human capital structure, and improve their R&D capabilities.

**Table 8:** Mechanism test at the human capital upgrading dimension: enhancing enterprises’ human capital level.

Variables	(1)	(2)	(3)	(4)
	High_edu	High_edu	Rdperson	Rdperson
Treat_post	0.008*	0.010**	0.035***	0.031***
	(0.005)	(0.005)	(0.004)	(0.004)
Age		−0.080***		0.012
		(0.023)		(0.020)
Lev		−0.023*		−0.025***
		(0.013)		(0.009)
Cash		−0.061***		0.003
		(0.017)		(0.011)
Roa		0.066***		0.023
		(0.024)		(0.014)
Kdensity		0.009**		0.007***
		(0.004)		(0.003)
Mshare		0.000		0.000
		(0.000)		(0.000)
Bigholder		−0.026		−0.010
		(0.027)		(0.019)
Growth		0.000*		−0.000
		(0.000)		(0.000)
Tbq		0.001		0.001
		(0.001)		(0.001)
YEAR	YES	YES	YES	YES
IND	YES	YES	YES	YES
Constant	0.158***	0.273***	0.034***	−0.073
	(0.004)	(0.085)	(0.003)	(0.062)
Observations	16,336	15,254	12,781	11,789
R-squared	0.112	0.110	0.416	0.371
Number of stkcd	2,356	2,349	2,332	2,323

The table reports test results of mechanism test at the human capital upgrading dimension: Enhancing Enterprises’ Human Capital Level. ***, **, * denote the significance of the respective coefficients at 1, 5, and 10% levels.

### 6.3 Capital investment dimension: enhancing enterprises’ capital investment level

To verify that intelligent manufacturing strategies incentivize firms to aggressively increase investment in both physical capital and intangible capital, this study follows the approach of Quan and Li (2022). Specifically, we measure firm-level capital investment (Devices) as the sum of the year-end book value of machinery and equipment plus the year-end book value of software assets. Meanwhile, we use enterprises’ robot ownership level to represent their utilization level of intelligent equipment.^2^

Based on Equation 1, the following Equations 7, 8 is constructed:

$${\text{Devices}}_{\mathrm{it}}=\alpha_{0}+\theta {\text{Treat}}_{i}\times {\text{Post}}_{t}+\beta X_{\mathrm{it}}+\mu_{i}+\gamma_{t}+\varepsilon_{\mathrm{it}}$$ (7)

$$\text{Robot}\_{\text{density}}_{\mathrm{it}}=\alpha_{0}+\theta {\text{Treat}}_{i}\times {\text{Post}}_{t}+\beta X_{\mathrm{it}}+\mu_{i}+\gamma_{t}+\varepsilon_{\mathrm{it}}$$ (8)

From the regression results in Table 9, it can be observed that intelligent manufacturing strategies increase enterprises’ capital investment (Devices). The regression coefficients of the interaction term (Treat×Post) with enterprises’ capital investment (Machine) and robot ownership (Robot_density) are 0.165 and 0.310 respectively, and both are significantly positive at the 1% level. These regression results verify the aforementioned mechanism: Intelligent manufacturing strategies promote intelligent manufacturing enterprises to increase capital investment and the use of industrial robot equipment, and significantly improve the labor productivity.

**Table 9:** Mechanism at the capital investment dimension: enhancing enterprises’ capital investment level.

Variables	Devices	Devices	Robot_density	Robot_density
Treat_post	0.218***	0.165***	0.348***	0.310***
	−0.06	−0.046	−0.037	−0.035
Age		0.680***		0.263
		−0.204		−0.2
Lev		1.182***		0.355***
		−0.133		−0.088
Cash		0.125		0.075
		−0.114		−0.095
Roa		0.890***		−0.322*
		−0.184		−0.166
Kdensity		0.616***		−0.005
		−0.033		−0.022
Mshare		−0.001*		−0.001
		−0.001		−0.001
Bigholder		0.058		−0.467***
		−0.238		−0.172
Growth		0.002***		0
		0		0
Tbq		−0.059***		0.009
		−0.01		−0.008
Year	Yes	Yes	Yes	Yes
Ind	Yes	Yes	Yes	Yes
Constant	18.678***	9.060***	1.224***	2.874***
	−0.04	−0.674	(0.023)	−1.082
Observations	10,444	9,547	6,911	6,291
R-squared	0.237	0.456	0.878	0.887
Number of stkcd	1,960	1,903	741	738

The table reports test results of mechanism at the capital investment dimension: Enhancing Enterprises’ Capital Investment Level. ***, **, * denote the significance of the respective coefficients at 1, 5, and 10% levels.

### 6.4 Mediating effect analysis

Based on the above regression results, we examine the mediating effect of intelligent manufacturing strategy on enterprises’ labor productivity following the procedure summarized by Wang et al. (2018). As shown in Table 10: ① All sequential tests are statistically significant, suggesting that the causal step approach performs better than other mediating effect methods (Wang et al., 2018); ② Intelligent manufacturing strategy significantly promotes enterprises’ labor productivity through alleviating financing constraints, upgrading human capital, and increasing capital expenditure, which supports the research hypotheses proposed in this paper.

**Table 10:** Results and inference of mediating effect tests.

Test procedure	①To examine the total effect *c* of the independent variable *treat_post* on the dependent variable *MPL*	②To test effect *a* of the independent variable *treat_post* on the mediating variable *M*; and effect *b* of the mediating variable *M* on the dependent variable *MPL* after controlling for the influence of the independent variable *treat_post*	③After controlling for the influence of the mediating variable *M*, test the direct effect *c′* of the independent variable *treat_post* on the dependent variable *v*	④ To conduct mediation effect inference by testing whether *ab* and *c′* have the same sign
Examine the relationship	Intelligent Manufacturing Strategy (*treat_post*)-Corporate Financing Constraint Index (*finan_bind*)- Enterprise Labor Productivity (*MPL*)			
Test results	*c* = 0.1534*** indicating a potential mediating effect	*a* = −0.0062*, *b* = −0.3929* both a and b are statistically significant, indicating that the indirect effect of finan_bind on MPL in the path where treat_post affects MPL is significant	*c*′ = 0.1526*** and the direct effect is significant, suggesting the existence of other mediators	ab and c′ have the same sign, indicating that finan_bind plays a partial mediating role in the impact of treat_post on MPL, the magnitude of the mediating effect is ab/c = 0.0159
Examine the relationship	Intelligent Manufacturing Strategy (*treat_post*)-Corporate Financing Constraint Index (*FC*)-Enterprise Labor Productivity (M*PL*)			
Test results	*c* = 0.1534*** indicating a potential mediating effect	*a* = −0.0173**, *b* = −0.9406*** both a and b are statistically significant, indicating that the indirect effect of FC on MPL through the path of treat_post affecting MPL is significant.	*c*′ = 0.1388*** and the direct effect is significant, suggesting the existence of other mediators	ab and c′ have the same sign, indicating that FC plays a partial mediating role in the impact of treat_post on MPL, the magnitude of the mediating effect is ab/c = 0.1061
Examine the relationship	Intelligent Manufacturing Strategy (*treat_post*)-R&D investment (*rd_invest*)-Enterprise Labor Productivity (M*PL*)			
Test results	*c* = 0.1534*** indicating a potential mediating effect	*a* = 0.3036**, *b* = 0.0307** both a and b are statistically significant, indicating that the indirect effect of rd_invest on MPL through the path of treat_post affecting MPL is significant	*c*′ = 0.1628*** and the direct effect is significant, suggesting the existence of other mediators	ab and c′ have the same sign, indicating that rd_invest plays a partial mediating role in the impact of treat_post on MPL, the magnitude of the mediating effect is ab/c = 0.0608
Examine the relationship	Intelligent Manufacturing Strategy (*treat_post*)-R&D innovation (*total_patent*)-Enterprise Labor Productivity (M*PL*)			
Test results	*c* = 0.1534*** indicating a potential mediating effect	*a* = 0.0824**, *b* = 0.1756*** both a and b are statistically significant, indicating that the indirect effect of total_patent on MPL through the path of treat_post affecting MPL is significant	*c*′ = 0.1387*** and the direct effect is significant, suggesting the existence of other mediators	ab and c′ have the same sign, indicating that total_patent plays a partial mediating role in the impact of treat_post on MPL, the magnitude of the mediating effect is ab/c = 0.0943
Examine the relationship	Intelligent Manufacturing Strategy (*treat_post*)-Corporate human capital (*high_edu*)-Enterprise Labor Productivity (M*PL*)			
Test results	*c* = 0.1534*** indicating a potential mediating effect	*a* = 0.0089***, *b* = 0.0821* both a and b are statistically significant, suggesting that the indirect effect of high_edu on MPL through the influence of treat_post is significant	*c*′ = 0.1541*** and the direct effect is significant, suggesting the existence of other mediators	ab and c′ have the same sign, indicating that high_edu plays a partial mediating role in the impact of treat_post on MPL, the magnitude of the mediating effect is ab/c = 0.0048
Examine the relationship	Intelligent Manufacturing Strategy (*treat_post*)-capital investment (*Machine*)-Enterprise Labor Productivity (*MPL*)			
Test results	*c* = 0.1534*** indicating a potential mediating effect	*a* = 0.1564***, *b* = 0.3704***, both a and b are statistically significant, suggesting that the indirect effect of Machine on MPL through the influence of treat_post is significant	*c*′ = 0.0840** and the direct effect is significant, suggesting the existence of other mediators	ab and c′ have the same sign, indicating that Machine plays a partial mediating role in the impact of treat_post on MPL, the magnitude of the mediating effect is ab/c = 0.3776
Examine the relationship	Intelligent Manufacturing Strategy (*treat_post*)-capital investment (*Robot*)-Enterprise Labor Productivity (*MPL*)			
Test results	*c* = 0.1534*** indicating a potential mediating effect	*a* = 0.2973**, *b* = 0.0549*, both a and b are statistically significant, suggesting that the indirect effect of Robot on MPL through the influence of treat_post is significant	*c*′ = 0.1312*** and the direct effect is significant, suggesting the existence of other mediators	ab and c′ have the same sign, indicating that Robot plays a partial mediating role in the impact of treat_post on MPL, the magnitude of the mediating effect is ab/c = 0.1064

The table reports results and inference of mediating effect tests. ***, **, * denote the significance of the respective coefficients at 1, 5, and 10% levels.

## 7 Further discussion

### 7.1 Regional differences: eastern region vs. central region vs. western region

Owing to disparities in historical development, geographical location, and policy orientation, China’s eastern, central, and western regions differ substantially in factor endowments, industrial structure, and institutional environment. These regional discrepancies profoundly shape firms’ production and operational decisions, thereby affecting their labor productivity. Given the pronounced gaps in economic foundations, industrial support, and resource endowments across the three regions, the impact of intelligent manufacturing policies on labor productivity is expected to exhibit significant regional heterogeneity. Benefiting from industrial clusters and sound infrastructure, the eastern region achieves higher efficiency in policy implementation.

In contrast, the western region features fragmented industrial clusters and relatively low intelligent equipment utilization compared with the eastern region, which indirectly constrains the magnitude of labor productivity improvement for local enterprises. Meanwhile, policies in the eastern region focus on innovation incentives to support high-end industrial development, whereas policies in the central region prioritize transformation subsidies and strongly promote the “robot replacement” campaign to facilitate traditional industrial upgrading. However, the western region is dominated by resource-based processing firms (e.g., coal and non-ferrous metal enterprises) with limited R&D demand, preventing intelligent manufacturing policies from effectively translating into labor productivity gains.

From the regression results in Table 7 below, it can be observed that the intelligent manufacturing strategy has a more significant impact on the labor productivity (MPL) in the eastern and central regions, while its impact on the MPL in the western region is not significant. This confirms that the differences in China’s regional environments affect enterprises’ labor productivity.

### 7.2 Difference in ownership attribute: state-owned enterprises vs. non-state-owned enterprises

Overall, the intelligent manufacturing strategy affects labor productivity in both state-owned enterprises (SOEs) and non-state-owned enterprises (non-SOEs), yet notable differences exist in the magnitude and mechanism of its impact. The strategy encourages SOEs to increase R&D investment and pursue technological innovation, thereby improving labor productivity. Nevertheless, owing to low innovation efficiency in some SOEs, the contribution of intelligent manufacturing policies to their labor productivity may remain insignificant. For non-SOEs, the intelligent manufacturing strategy significantly boosts innovation output. Specifically, it enables non-SOEs to access relevant policy support and promotes the development and application of cutting-edge technologies.

The intelligent manufacturing strategy has a direct impact on the labor productivity of non-SOEs: through replacing manual labor with technology and optimizing processes to reduce costs, it greatly improves the production efficiency. Moreover, this impact is often more significant in non-SOEs. The reason lies in that non-SOEs make decisions based on market conditions—they have higher decision-making efficiency and a stronger market-oriented orientation, which are more conducive to the rapid implementation of technological upgrading.

As shown in the regression results in Table 11 the intelligent manufacturing strategy has a statistically significant effect on both SOEs and non-SOEs. This indicates that intelligent manufacturing policies significantly improve labor productivity in both types of firms.

**Table 11:** Regression results of regional differences & property right attribute differences.

Variables	(1)	(2)	(3)	(4)	(5)
	MPL	MPL	MPL	MPL	MPL
	Eastern China	Central China	Western China	SOEs	non-SOEs
Treat_post	0.150***	0.146**	0.118	0.124***	0.154***
	(0.034)	(0.062)	(0.072)	(0.047)	(0.033)
Age	0.887***	0.949***	0.613	0.573*	0.827***
	(0.157)	(0.354)	(0.493)	(0.302)	(0.160)
Lev	1.253***	1.139***	1.067***	1.056***	1.167***
	(0.104)	(0.151)	(0.193)	(0.149)	(0.098)
Cash	0.573***	0.452***	1.092***	0.411***	0.691***
	(0.086)	(0.166)	(0.200)	(0.134)	(0.084)
Roa	2.270***	2.409***	2.063***	2.116***	2.274***
	(0.159)	(0.357)	(0.367)	(0.235)	(0.161)
Kdensity	0.027	−0.003	0.026	0.075*	0.002
	(0.021)	(0.043)	(0.043)	(0.042)	(0.018)
Mshare	−0.001***	−0.000	−0.006***	−0.003	−0.001**
	(0.001)	(0.001)	(0.002)	(0.005)	(0.000)
Bigholder	−0.113	0.373	−0.322	0.278	−0.164
	(0.173)	(0.329)	(0.388)	(0.262)	(0.180)
Growth	0.005***	0.006***	0.005***	0.006***	0.005***
	(0.000)	(0.000)	(0.000)	(0.000)	(0.000)
Year	Yes	Yes	Yes	Yes	Yes
Ind	Yes	Yes	Yes	Yes	Yes
Constant	17.682***	17.861***	18.714***	18.505***	17.944***
	(0.478)	(0.980)	(1.423)	(0.945)	(0.458)
Observations	10,467	2,993	2,109	4,475	10,803
R-squared	0.564	0.510	0.504	0.431	0.592
Number of stkcd	1,655	417	294	616	1,829

The table reports regression results of regional differences & property right attribute differences. ***, **, * denote the significance of the respective coefficients at 1, 5, and 10% levels.

### 7.3 Industry technology update speed difference: fast technology update speed vs. slow technology update speed

Technological iteration speed reflects a firm’s ability to track and adopt cutting-edge technologies. Intelligent manufacturing firms typically maintain a relatively high pace of technological iteration to sustain their competitive advantages. Firms with faster technological iteration can better keep pace with, or even lead, advances in cutting-edge technologies. As such, the impact of intelligent manufacturing policies on labor productivity is relatively weaker for these firms.

However, for firms with slower technological iteration, the strategic guidance and support provided by intelligent manufacturing policies may exert a stronger effect on labor productivity. This is because policy implementation enables firms with slow technological iteration to timely obtain information about market dynamics and peers’ technological progress. In turn, this helps them improve production and operational efficiency, conduct more innovation activities, and better cope with intensified market competition. Accordingly, intelligent manufacturing policies exhibit significantly heterogeneous effects on labor productivity across firms with different speeds of technological iteration.

Following the approach of Shen et al. (2024), this study classifies manufacturing industries including computer, communication, and other electronic equipment manufacturing, as well as electrical machinery manufacturing, as industries with fast technological iteration. All other manufacturing industries are classified as those with slow technological iteration, and this classification is captured by a dummy variable (techupdate). The regression results are reported in Table 12. Consistent with our expectations, the results show that the positive effect on labor productivity (MPL) is stronger in industries with slow technological iteration. This indicates that firms in slow-iteration industries are more responsive to intelligent manufacturing policies, which therefore exert a more pronounced effect in boosting labor productivity in these industries.

**Table 12:** Regression results of differences in industry technological iteration speed and enterprise scale differences.

Variables	(1)	(2)	(3)	(4)
	MPL	MPL	MPL	MPL
	Industry technology update speed		Enterprise scale	
	Fast	Slow	Large-scale	Small-scale
Treat_post	0.052	0.110***	0.159***	−0.046
	(0.096)	(0.030)	(0.028)	(0.056)
Age	1.101***	0.779***	0.897***	−0.015
	(0.289)	(0.151)	(0.144)	(0.312)
Lev	1.350***	1.055***	1.122***	−0.024
	(0.194)	(0.081)	(0.068)	(0.126)
Cash	0.730***	0.672***	0.606***	0.390**
	(0.160)	(0.081)	(0.071)	(0.170)
Roa	1.773***	2.356***	2.231***	0.661***
	(0.269)	(0.167)	(0.140)	(0.192)
Kdensity	−0.005	0.026	0.028*	−0.040**
	(0.030)	(0.018)	(0.017)	(0.019)
Mshare	−0.003***	−0.001**	−0.002***	0.001
	(0.001)	(0.000)	(0.000)	(0.001)
Bigholder	−0.151	0.021	−0.091	−0.107
	(0.296)	(0.160)	(0.140)	(0.194)
Growth	0.005***	0.006***	0.005***	0.003***
	(0.000)	(0.000)	(0.000)	(0.000)
Tbq	−0.045***	−0.024***	−0.020***	0.024***
	(0.014)	(0.007)	(0.006)	(0.007)
Year	Yes	Yes	Yes	Yes
Ind	Yes	Yes	Yes	Yes
Constant	18.695***	15.896***	19.756***	11.168***
	(1.611)	(1.235)	(1.015)	(1.254)
Observations	3,948	11,296	13,839	1,405
R-squared	0.588	0.534	0.545	0.355
Number of stkcd	654	1,764	2,236	488

The table reports regression results of differences in industry technological iteration speed & enterprise Scale differences. ***, **, * denote the significance of the respective coefficients at 1, 5, and 10% levels.

### 7.4 Differences in enterprise scale: large scale vs. small scale

The scale attribute of an enterprise directly affects the effectiveness of intelligent manufacturing policies in promoting enterprise labor productivity. Intelligent manufacturing policies may exert different degrees of impact on enterprises of varying scales. Typically, large-scale enterprises possess abundant capital and more mature innovation mechanisms. They also have accumulated a wealth of technical talents, more sophisticated marketing and R&D departments. Relying on intelligent manufacturing policies, these enterprises can accelerate their intelligent transformation, continuously improve production and operation efficiency, enhance technological innovation capabilities.

Therefore, compared with small and medium-sized enterprises (SMEs), large-scale enterprises are better able to absorb and utilize the policy dividends, thus more effectively improve their labor productivity. In this paper, large-scale enterprises are defined in accordance with the classification standards of the National Bureau of Statistics. Specifically, enterprises with an annual operating income exceeding 400 million yuan (about 56.72 million US dollars) are classified as large-scale enterprises, this classification is represented by a dummy variable (scale).

The regression results are shown in Table 12. These results indicate that the intelligent manufacturing policies on the labor productivity of intelligent manufacturing enterprises is more significant in large-scale enterprises. The comparative advantages of large-scale enterprises in technology and capital enable them to better leverage the positive effects, thereby forming an effective incentive for the improvement of enterprise labor productivity.

## 8 Research conclusions and policy recommendations

This study finds that the intelligent manufacturing strategy significantly improves firm labor productivity. This conclusion holds after a series of robustness checks, including the parallel trend test, placebo test, and propensity score matching (PSM). Mechanism analysis shows that the intelligent manufacturing strategy enhances labor productivity by boosting innovation capacity, optimizing human capital structure, and increasing capital investment. Heterogeneity analysis indicates that the policy effect is more pronounced in the eastern and central regions, among large-scale firms, and in industries with slow technological iteration. This paper verifies the effects and underlying mechanisms of China’s intelligent manufacturing strategy on firm labor productivity, offering Chinese experience for the promotion of intelligent manufacturing policies in other countries and regions.

Based on the aforementioned research, this paper puts forward policy recommendations from the following aspects: (1) Strengthen targeted policy support based on the policy efficiency analysis. Optimize the allocation of the “Special Fund for Intelligent Manufacturing Transformation,” focus on supporting leading enterprises with high subsidy efficiency to build industrial chain collaboration platforms, and provide gradient subsidies according to the scale of enterprise transformation and the expected effect of labor productivity improvement, so as to improve the efficiency of policy funds. (2) Promote technology R&D and innovation based on the mechanism of innovation-driven labor productivity improvement. Increase support for key intelligent manufacturing technologies that have been verified to significantly promote labor productivity, encourage enterprises to cooperate with research institutions to tackle technical bottlenecks, and focus on breaking through core technologies closely related to the three mechanism paths (innovation capacity, human capital structure, capital investment) to provide targeted technical support. (3) Establish a targeted talent guarantee mechanism based on the path of human capital structure optimization. Formulate preferential policies for introducing high-end intelligent manufacturing talents and provide research start-up funds and living subsidies; improve the talent training system, focus on carrying out skill training for existing employees in big data, AI and other fields closely related to intelligent production, and improve the matching degree between human capital and intelligent manufacturing needs.

## Data Availability

The data underlying this study are available from the CSMAR, WIND, and CNRDS databases. Readers can access these datasets through the following websites: https://data.csmar.com/, https://www.wind.com.cn/, and https://www.cnrds.com/.

## Footnotes

1 This paper measures the level of regional digital infrastructure construction using the ratio of the number of internet broadband access users to the total number of urban residents in the city where the enterprise is located.

2 Drawing on data from the International Federation of Robotics (IFR), we use the stock of robots used in the manufacturing industry (robot) as a proxy variable for enterprises’ intelligent equipment.
