# Supplementary material for: The impact of intelligent manufacturing strategy on enterprise labor productivity: evidence from a quasi-natural experiment in China
Source: Front Artif Intell. 2026 May 26;9:1775528. doi: 10.3389/frai.2026.1775528 (PMC13246367; doi:10.3389/frai.2026.1775528)
Supplement: Supplementary file 1 [file Data_Sheet_1.docx]

**Addressing Endogeneity**

Although the intelligent manufacturing policy is guided by national strategy, and it is difficult for enterprises to directly intervene in the promulgation of the policy, the implementation of the intelligent manufacturing policy ultimately depends on the degree of implementation at the enterprise level. If the intelligent manufacturing policy can improve labor productivity, both enterprises with high and low labor productivity would have incentives to carry out intelligent transformation under the policy guidance. Considering the potential omitted variable bias, the baseline regression results of this paper may be subject to endogeneity concerns. Therefore, this paper adopts the instrumental variable method to mitigate endogeneity in the model.

Specifically, following the practice of Quan and Li (2022), this paper selects the number of employees in the telecommunications and other information transmission services in the enterprise’s region in 2003 as the instrumental variable for the intelligent manufacturing policy. On the one hand, the implementation of intelligent manufacturing policies and intelligent transformation by enterprises cannot be separated from the support of regional network infrastructure. Under the impact of the intelligent manufacturing policy, the input in telecommunications and other information transmission services directly affects the popularization and development of Internet technology in the region, which meets the relevance requirement of instrumental variables. On the other hand, the stock of regional development level of telecommunications and other information transmission services can hardly directly affect current labor productivity, which satisfies the exogeneity requirement of instrumental variables, indicating that the instrumental variable selected in this paper is valid.

Therefore, referring to Quan and Li (2022), this paper uses the ratio of regional industry employment to national manufacturing employment multiplied by the number of employees in telecommunications and other information transmission services in the region in 2003 as the instrumental variable for intelligent manufacturing.

The results of the two-stage least squares (2SLS) estimation are reported in the attached table.The first-stage regression shows that the selected instrumental variable is highly positively correlated with the intelligent manufacturing policy variable, which is consistent with expectations. The first-stage F-statistics are all greater than 10, and the K-P F-statistics also exceed the critical values, rejecting the null hypothesis of weak instruments. These results confirm that the instrumental variable employed in this paper is valid.In the second-stage regressions, all coefficients are significantly positive at the 1% level. After addressing endogeneity concerns, the promoting effect of intelligent manufacturing policy implementation on enterprise labor productivity remains robust, and the main findings of this paper are unchanged.

**Table Instrumental Variable Regression**

|  | **(1)** | **(2)** | **(3)** | **(4)** |
| --- | --- | --- | --- | --- |
| **Variable** | **MPL** | **MPL** | **MPL** | **MPL** |
| Treat×post | 0.776*** | 0.623*** | 0.622*** | 0.754*** |
|  | (0.166) | (0.146) | (0.144) | (0.149) |
| Age |  | 0.753*** | 0.735*** | 0.809*** |
|  |  | (0.139) | (0.143) | (0.150) |
| Lev |  | 1.272*** | 1.253*** | 1.107*** |
|  |  | (0.089) | (0.090) | (0.084) |
| Cash |  | 0.816*** | 0.784*** | 0.671*** |
|  |  | (0.084) | (0.085) | (0.079) |
| Roa |  | 2.129*** | 2.151*** | 2.394*** |
|  |  | (0.138) | (0.140) | (0.141) |
| Kdensity |  |  | 0.001 | 0.024 |
|  |  |  | (0.019) | (0.017) |
| Mshare |  |  | -0.001*** | -0.002*** |
|  |  |  | (0.000) | (0.000) |
| Bigholder |  |  | -0.014 | -0.175 |
|  |  |  | (0.160) | (0.145) |
| Growth |  |  |  | 0.006*** |
|  |  |  |  | (0.000) |
| Tbq |  |  |  | -0.033*** |
|  |  |  |  | (0.007) |
|  | **First-stage regression** | | |  |
| IV: Number of employees in telecommunications in 2003 | 1.597^***^ | 1.545^***^ | 1.798^***^ | 1.847^***^ |
|  | (0.086) | (0.086) | (0.097) | (0.103) |
| Control | YES | YES | YES | YES |
| First-stage F-statistic | 21.86 | 101.82 | 64.11 | 137.54 |
| YEAR | YES | YES | YES | YES |
| Firm | YES | YES | YES | YES |
| IND | YES | YES | YES | YES |
| Obs | 15881 | 15881 | 15394 | 14797 |
| Adjusted R^2^ | -0.089 | 0.095 | 0.096 | 0.183 |
| KP F-statistic | 309.677 | 308.044 | 303.24 | 296.952 |
